# Illinois State Veterinary Medical Association

**Published:** 1897-04

**Authors:** Albert Babb

**Affiliations:** Secretary


Secretary.

### ILLINOIS STATE VETERINARY MEDICAL ASSOCIATION.

THE semi-annual meeting was held at Leland Hotel, Springfield, Ill., February 17, 1897. The meeting was called to order by the President, Dr. M. R. Trumbower, at 10 A.M. On roll-call by the Secretary the following members responded: A. G. Alverson, Albert Babb, A. H. Baker, L. Campbell, James Henderson, J. F. Pease, H. G. Pyle, E. L. Quitman, G. G. Ratz, C. E. Sayre, M. R. Trumbower, N. P. Whitmore, and W. H. Welch. The minutes of the previous meeting were read and approved.

On motion, the talk on "State Work," by Dr. M. R. Trumbower, was postponed till afternoon. Dr. J. F. Pease read his paper on "Study of Odontomes," which was very interesting and liberally discussed. The Society then listened to the very instructive essay of Dr. W. H. Welch on the subject "Milk Sickness." On motion, the ensuing discussion was closed. On motion, a vote of thanks was tendered to the essayists for their productions.

On motion, a committee of three, consisting of Drs. Albert Babb, A. H. Baker, and J. F. Pease, was appointed to go before the House Committee of Live-stock and Dairying, before which our veterinary bill is to come this afternoon. On motion, Drs. M. R. Trumbower and A. G. Alverson were added to the committee.

On motion, the Society adjourned until 4.30 P.M. The Society reconvened at the appointed hour and listened to the report of the committee through its chairman, Dr. Albert Babb. The House Committee of Live-stock and Dairying postponed action on the veterinary bill for one week.

On motion, the rules were suspended for the time being and the following gentlemen elected to membership by acclamation: Dr. G. G. Grundy (C. V. C., 1890), Morrisonville, Ill.: vouchers, Drs. Albert Babb and N. P. Whitmore; E. J. List (C. V. C., 1892), Havana, Ill.: vouchers, Drs. Albert Babb and M. R. Trumbower.

The President appointed a committee of three, consisting of Drs. Welch, Alverson, and Quitman, to draft resolutions in regard to the death of Dr. J. W. Parkinson, late of El Paso, Ill. They made the following report:

WHEREAS, The all-wise Providence has seen fit to remove from our midst our professional brother and former co-laborer, J. W. Parkinson, late of El Paso, Ill., an enterprising and conscientious veterinarian, whose studious and gentlemanly qualities placed him in the front rank of our profession; be it hereby

*Resolved*, That we, the Illinois State Veterinary Medical Association, deeply deplore his loss to the Association and to the profession at large, and sincerely sympathize with his family in their bereavement.

*Resolved*, That these resolutions be spread upon the minutes of this Society and that a copy be forwarded to the family of the deceased.

Dr. Trumbower then gave the Society a very entertaining and instructive discourse on "State Work."

On motion, the Society adjourned, to meet in Chicago in November, at the call of the President.

ALBERT BABB, A.B., M.D.C.,
Secretary.

